# Digital Health Technology for Real-World Clinical Outcome Measurement Using Patient-Generated Data: Systematic Scoping Review

**DOI:** 10.2196/46992

**Published:** 2023-10-11

**Authors:** Evelyn Pyper, Sarah McKeown, Jamie Hartmann-Boyce, John Powell

**Affiliations:** 1 Department for Continuing Education University of Oxford Oxford United Kingdom; 2 Nuffield Department of Primary Care Health Sciences University of Oxford Oxford United Kingdom; 3 Department of Health Promotion and Policy University of Massachusetts Amherst Amherst, MA United States

**Keywords:** real-world evidence, real-world data, digital tools, digital health, digital biomarkers, patient-generated health data, mobile health, mHealth, wearables, digital health management, clinical intervention, electronic health record, health outcomes, mobile phone

## Abstract

**Background:**

Digital health technologies (DHTs) play an ever-expanding role in health care management and delivery. Beyond their use as interventions, DHTs also serve as a vehicle for real-world data collection to characterize patients, their care journeys, and their responses to other clinical interventions. There is a need to comprehensively map the evidence—across all conditions and technology types—on DHT measurement of patient outcomes in the real world.

**Objective:**

We aimed to investigate the use of DHTs to measure real-world clinical outcomes using patient-generated data.

**Methods:**

We conducted this systematic scoping review in accordance with the Joanna Briggs Institute methodology. Detailed eligibility criteria documented in a preregistered protocol informed a search strategy for the following databases: MEDLINE (Ovid), CINAHL, Cochrane (CENTRAL), Embase, PsycINFO, ClinicalTrials.gov, and the EU Clinical Trials Register. We considered studies published between 2000 and 2022 wherein digital health data were collected, passively or actively, from patients with any specified health condition outside of clinical visits. Categories for key concepts, such as DHT type and analytical applications, were established where needed. Following screening and full-text review, data were extracted and analyzed using predefined fields, and findings were reported in accordance with established guidelines.

**Results:**

The search strategy identified 11,015 publications, with 7308 records after duplicates and reviews were removed. After screening and full-text review, 510 studies were included for extraction. These studies encompassed 169 different conditions in over 20 therapeutic areas and 44 countries. The DHTs used for mental health and addictions research (111/510, 21.8%) were the most prevalent. The most common type of DHT, mobile apps, was observed in approximately half of the studies (250/510, 49%). Most studies used only 1 DHT (346/510, 67.8%); however, the majority of technologies used were able to collect more than 1 type of data, with the most common being physiological data (189/510, 37.1%), clinical symptoms data (188/510, 36.9%), and behavioral data (171/510, 33.5%). Overall, there has been real growth in the depth and breadth of evidence, number of DHT types, and use of artificial intelligence and advanced analytics over time.

**Conclusions:**

This scoping review offers a comprehensive view of the variety of types of technology, data, collection methods, analytical approaches, and therapeutic applications within this growing body of evidence. To unlock the full potential of DHT for measuring health outcomes and capturing digital biomarkers, there is a need for more rigorous research that goes beyond technology validation to demonstrate whether robust real-world data can be reliably captured from patients in their daily life and whether its capture improves patient outcomes. This study provides a valuable repository of DHT studies to inform subsequent research by health care providers, policy makers, and the life sciences industry.

**Trial Registration:**

Open Science Framework 5TMKY; https://osf.io/5tmky/

## Introduction

### Background

Digital health technology (DHT) has opened the door to novel processes and insights that previously would not have been attainable through traditional health system infrastructure, including virtual care, new diagnostic and disease surveillance mechanisms, and real-time patient self-monitoring. Beyond their use as interventions, DHTs serve as an important vehicle for the collection of real-world health-related data—including health history, symptoms, biometric data, treatment history, and lifestyle choices. When this information is created, recorded, or gathered by or from patients or their care partners to help address a health concern, it is referred to as patient-generated health data (PGHD) [1]. By shifting the responsibility and ownership of data collection to the patient, individuals may be more empowered to understand their own health and even contribute their information to care providers or research studies.

### Digital Measurement

For clinicians and researchers, PGHD from digital technologies can serve to increase the number of observations for each patient and even capture information that would not otherwise be found in medical records or health care claims, such as patient-reported outcomes or experience measures. Data can be collected *actively*, requiring more action by a human intermediary (eg, survey); *passively*, whereby the need for human participation is minimal (eg, sensor); or through the *hybrid* collection of both active and passive metrics (eg, multimodal measures) [2]. In routine clinical care, digital measures can be used to drive earlier diagnosis, inform care decisions, assess treatment adherence, and monitor real-time safety and performance. In clinical research, digital measurement can be used to inform novel end points for hard-to-measure conditions; enable more objective and precise screening for inclusion and exclusion in clinical trials; and assess medication adherence, safety, or efficacy [2].

### DHT in Clinical Research

The use of DHTs in clinical trials has grown substantially since 2000 at a rate of 34% per year, with applications spanning all phases of clinical research [3]. Beyond trial optimization and endpoint selection, DHTs—defined as systems that use computing platforms, connectivity, software, or sensors for health care and related uses—offer the life sciences sector a way to gather clinical insights on patients outside of traditional clinical trials [4]. This real-world evidence is increasingly used by the pharmaceutical industry to assess patient outcomes, support regulatory approvals, study more diverse patients or rare disease populations, link treatment pricing to effectiveness, and streamline the use of resources. Regulators and payers are also increasingly looking to manufacturers to demonstrate the ongoing value of their products using real-world evidence. The data used to derive this evidence, real-world data (RWD), can include clinical and economic outcomes, patient-reported measures, and quality of life and has traditionally come from sources such as electronic medical records, claims databases, and patient registries [5]. DHTs introduced a novel means for RWD capture that, through their proximity to the patient, supports the study of patient behavior (eg, adherence and compliance), comorbidities, and contextual information [6].

### Digital Biomarkers

DHTs enable continuous, real-time, and often passive and unobtrusive measurement of behavior and physiology in the real world, thereby generating a high volume of data [7]. Relatedly, the use of objective, quantifiable physiological and behavioral data can serve as *digital biomarkers* to diagnose disease, monitor biological processes, and predict outcomes [8]. Sensitive digital biomarkers play an increasingly important role in personalized prediction and precision medicine [9,10].

Digital biomarkers for personalized mental health diagnosis and treatment—where DHTs can help fill gaps in measures that are often challenging to measure in episodic clinic visits—represent a burgeoning body of research [11-14]. Beyond neuropsychiatry, reviews of the literature on DHT-based measurements have been conducted in many clinical areas including neurodegenerative disease [15], asthma [16], and surgical care [17]. Reviews have also been conducted for specific data-collection approaches, such as speech-based digital biomarkers [18], electronic patient-reported outcome measurements [19], and automated-entry PGHD [20]. However, to our knowledge—informed by database searches including PROSPERO, PubMed, and the Cochrane Database—there have not yet been any comprehensive reviews examining real-world outcomes from DHTs across all years, geography, and diseases.

In this scoping review, we investigated the use of DHTs to measure real-world clinical outcomes using patient-generated data. This research will serve to summarize and map the evidence in this rapidly growing and evolving area of research and to identify knowledge gaps that could inform future research, including subsequent systematic reviews. To present a comprehensive picture of DHTs for RWD collection, we systematically reviewed and identified all relevant literature across a wide range of diseases, geographic regions, DHT types, clinical outcomes, and analytical applications. Within this overarching objective, we explored several key research questions outlined in Textbox 1.

**Key research questions.**

1. How has the nature of the publications within this literature changed over time?
2. For what diseases and therapeutic areas are clinical outcomes being measured using digital health technologies (DHTs)?
3. Does the proportion of studies involving active, passive, or hybrid data collection differ across therapeutic areas?
4. How has the use of different DHTs for real-world clinical outcome measurement changed over time?
5. How do different types of data intersect with various analytical applications?
6. What is the relationship between the different data types used within the same studies?

## Methods

### Study Design

We conducted a scoping review in accordance with the guidance provided by the Joanna Briggs Institute Reviewer Manual [21]. The review follows the PRISMA-ScR (Preferred Reporting Items for Systematic Reviews and Meta-Analyses extension for Scoping Reviews) checklist (Multimedia Appendix 1) [22]. We registered the protocol in the Open Science Framework on February 15, 2021 (registration: 5TMKY) [23].

### Eligibility Criteria

We developed detailed inclusion and exclusion criteria (Multimedia Appendix 2) to guide reviewer decision-making throughout the study screening, full-text review, and selection. A summary of key eligibility criteria is presented in Textbox 2.

**Summary of the inclusion and exclusion criteria.**

**Inclusion criteria**

- Studies published between 2000 and 2022
- Primary studies (both experimental and observational) with quantitative data
- Participants with any health condition, so long as the population is well defined and the disease or condition is clearly stated
- Digital health technology (DHT) used for the collection of data on an accompanying clinical intervention or for the measurement of any clinical outcome or endpoint
- DHT-collected data that (1) are exclusively patient generated (active or passive measurement, not requiring clinician involvement) and (2) include health-related data (eg, biometrics, digital biomarkers, and clinical outcomes)
- DHT “connected” to the internet or via Bluetooth, a mobile app, or a USB device
- Clinical outcomes clearly identified (“clinical outcomes” here refers to any measurable changes in health or quality of life that result from care, encompassing all types of clinical measurements, including biomarkers and end points)

**Exclusion criteria**

- Studies published before the year 2000
- Purely qualitative studies, reviews, commentaries, editorials, studies without original data, or studies focused only on technology validation
- DHT only used as an intervention or exclusively for study recruitment and retention
- Clinical interventions not focused on individual-level care, such as public health interventions encompassing population-wide health promotion and disease prevention efforts (eg, condition-agnostic programming for a healthy diet and physical activity)

### Search Strategy

We performed an initial search of MEDLINE (PubMed) to identify relevant articles and assess their index terms and the text words or “keywords” contained in the titles and abstracts. The search engines Google and Google Scholar were also used to iteratively search for relevant published and unpublished information that could provide context and insight into terminology. We used the identified index terms and keywords to inform a full search strategy for all included databases: MEDLINE (Ovid), CINAHL, Cochrane (CENTRAL), Embase, PsycINFO, ClinicalTrials.gov, and the EU Clinical Trials Register. We first developed a search strategy for MEDLINE (Ovid), validated it by a specialized librarian, and then translated it for use in the other databases (Multimedia Appendix 3).

We included studies from any country; however, only those published in English were considered. Given the use of DHT in clinical trials has grown dramatically since 2000 [3], studies published from January 1, 2000, were included. We conducted the primary search of all the included databases on February 14, 2021. Given the long time frame required for the screening, full-text review, and extraction of a scoping review of this size, we conducted a search update to ensure the inclusion of all relevant, up-to-date studies. We carried out this updated search of MEDLINE (Ovid) on July 31, 2022.

### Study Selection

All identified citations were collated and uploaded into the reference manager Mendeley (Elsevier), and duplicates were removed. We imported the resulting collection of citations into the web-based systematic review management system Covidence (Veritas Health Innovation), which detected additional duplicates. Two independent reviewers piloted the eligibility criteria on a random sample of 25 titles and abstracts and discussed discrepancies, clarified concepts, and made any necessary modifications to the eligibility criteria. Throughout the study selection process, any disagreement between reviewers where a consensus could not be reached was resolved by an additional reviewer. We screened titles and abstracts against the predefined eligibility criteria (998/7308, 13.66% screened by 2 independent reviewers), and those identified as relevant were retrieved in full and imported into Covidence. Full-text articles of the selected citations were then reviewed for inclusion based on the eligibility criteria. We recorded the reasons for exclusion of full-text studies that did not meet the inclusion criteria.

### Data Extraction and Analysis

We conducted data extraction using a predefined data extraction form in Microsoft Excel after developing and piloting a draft extraction form for 10 studies. The final data extraction form included fields on *publication* (country and publication type), *participants* (disease or condition and therapeutic area [TA]), *study details* (number of participants, study objectives, study design, and what is being evaluated), DHT (DHT type, passive or active collection, and type of data collected), outcomes measured by the DHT, analytical applications of the study, and whether the use of artificial intelligence (AI) or machine learning was mentioned. Although there is no single way to categorize by TA, we took an inductive, bottom-up approach (using the specific disease or condition as a starting point) to search for, confirm, and assign medical specialty categories consistent with the language used by clinical researchers and the life sciences industry. Moreover, as part of this work—given the wide array of DHT types and applications, along with the lack of a universally accepted classification framework—we have outlined the key terms and concepts as used for this study (Table 1). We formulated an initial list based on the work of Coravos et al [2] and subsequently adapted it as publications were included in the review.

**Table 1:** Key terms and concepts.

Concept			Description
**DHT^a^ type**			
	Smartphone or mobile app	Application software designed to run on a mobile device, such as a smartphone	
	Assessments via a mobile platform	Non–app-based measurements on a mobile phone (eg, ecological momentary assessments)	
	Mobile phone (other)	Any other mobile phone–based system for outcome measurement; often used when the information provided is insufficient to classify the type of mobile phone–based DHT (where more information is provided, examples include mobile phone–based video calling, SMS text messaging, or web platform use).	
	Wearable	Sensors and devices that can be worn unobtrusively on the user’s body (eg, wrist-worn activity monitors or smart clothing)	
	Implantable	Sensors and devices partly or totally introduced into the human body; often refers to implanting on the skin (eg, implantable glucose sensor)	
	Ingestible	Sensors embedded in a medication that, when it interacts with stomach acid, transmits to a patch sensor worn over the abdomen, monitoring when a pill was taken [2]	
	Electronic medication monitors	Sensors integrated into the packaging of medicines (also called “smart packs”) to record when the drug was administered and deliver automatic reminders to take a medication [2].	
	Environmental sensors	Sensors or networks of sensors in the patient’s environment (eg, at home) that measure environmental factors or behaviors, such as full-body 3D motion capture.	
	Other parameter-specific biosensors	Any sensors that (1) do not fall into one of the other specified sensor categories and (2) are designed to measure specific biological parameters (eg, electrocardiogram, pulse oximeter, or thermometer)	
	Web-based platform	An internet- or website-based platform used to collect information	
	Software	Programs enabling a computer to perform a specific task, not including mobile app software	
	Other	Any other types of DHT that do not fall within one of the other 11 categories	
**Analytic applications**			
	Descriptive	*What happened?* The use of patient data to characterize the experiences or outcomes of a patient or group	
	Diagnostic	*Why did it happen?* The use of patient data to determine why particular experiences or outcomes are occurring (eg, disease diagnosis)	
	Predictive	*What might happen next?* The use of patient data to forecast potential future outcomes (eg, prediction of heart attack or relapse)	
	Prescriptive	*If X happens, then what?* The use of patient data to recommend an action based on a prediction (eg, if specific outcomes are observed, medication dose should be increased)	

^a^DHT: digital health technology.

## Results

### Selection and Characteristics of Sources of Evidence

**Figure 1:**
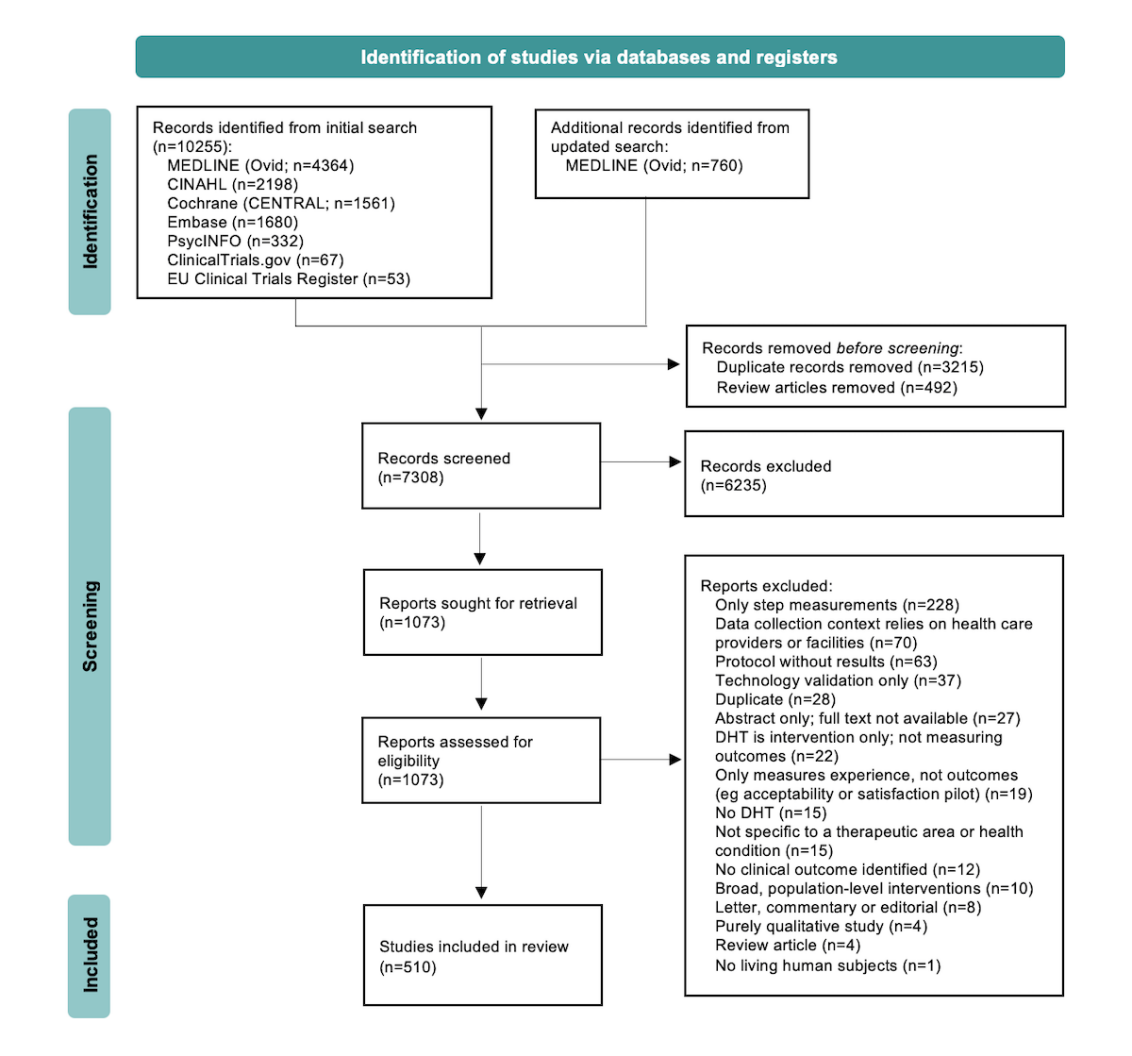
PRISMA (Preferred Reporting Items for Systematic Reviews and Meta-Analyses) flow diagram. DHT: digital health technology.

The flow of studies through the review process is shown in Figure 1. The search of databases and registers yielded 11,015 publications, of which 760 came from the updated search of MEDLINE (Ovid) in July 2022. After removing duplicates (n=3215) and review articles (n=492), the remaining 7308 articles underwent title and abstract screening, which excluded 6235 articles. The dominant reasons for exclusion at this stage pertained to study type (commentary, editorial, or study without original data); collection context (relying on health care providers or facilities); and population (healthy population or no defined patient population or TA). We retrieved full-text publications for the remaining 1073 articles and assessed them for eligibility. To separate studies of interest from the large pool of studies that used only a pedometer or accelerometer without being combined with other DHT measures, 228 (21.25%) of the 1073 articles that only captured step measurements were excluded. The next most common reasons for exclusion were studies where the data-collection context relied on health care providers or facilities (ie, not exclusively patient generated: 70/1073, 6.52%); published protocols without results available (63/1073, 5.87%); and studies that only validated the technology itself, rather than the application of that technology to measure clinical outcomes (37/1073, 3.45%). After screening and full-text review, 510 studies were included in the scoping review. Of the 510 included studies, 112 (22%) came from the updated search, representing the more recent articles published between February 2021 and July 2022.

Table 2 summarizes the characteristics of the included studies. Most publications were journal articles (413/510, 81%); however, conference abstracts (82/510, 16.1%), clinical trial protocols (6/510, 1.2%), dissertations (5/510, 1%), and research forum abstracts (4/510, 0.8%) were also included. Most articles originated from research conducted in North America (251/510, 49.2%), specifically the United States (243/510, 47.6%; see Figure S1 in Multimedia Appendix 4 for the geographical distribution of included studies by country of origin). Studies were published as early as 2003; however, the majority were published in the past 5 years (355/510, 69.6%). The included studies covered a variety of TAs, with the top 3 most common being mental health and addictions (111/510, 21.8%), neurology or central nervous system (CNS; 78/510, 15.3%), and cardiovascular (71/510, 13.9%). The most common type of DHT, smartphone or mobile apps, was reflected in approximately half of all the studies (250/510, 49%), followed by wearables (164/510, 32.2%), parameter-specific biosensors (72/510, 14.1%), and other mobile phone–based systems (62/510, 12.2%). Although many studies have involved multiple digital tools, the majority involved only 1 DHT (346/510, 67.8%) and did not evaluate any other intervention beyond the DHT itself (352/510, 69%). There was a fairly even split between studies where data were collected passively (189/510, 37.1%), actively (170/510, 33.3%), or through a hybrid approach (151/510, 29.6%). The information measured by each DHT was coded into one or more of 16 different data types, of which the most common were physiological data (189/510, 37.1%), clinical symptoms data (188/510, 36.9%), behavioral data (171/510, 33.5%), and physical activity data (165/510, 32.4%). Most articles did not apply AI (449/510, 88%) and leveraged the data collected for descriptive analytical applications (380/510, 74.5%).

**Table 2:** Summary of characteristics of included studies (n=510).

Characteristics		Studies, n (%)
**Continent**		
	North America	251 (49.2)
	Europe	130 (25.5)
	Not specified	71 (13.9)
	Asia	26 (5.1)
	Multicontinental	14 (2.7)
	Australia and Oceania	11 (2.2)
	Africa	7 (1.4)
**Publication year**		
	2003-2007	7 (1.4)
	2008-2012	27 (5.3)
	2013-2017	121 (23.7)
	2018-2022	355 (69.6)
**Publication type**		
	Journal article	413 (81)
	Conference abstract	82 (16.1)
	Clinical trial protocol	6 (1.2)
	Dissertation	5 (1)
	Research forum abstract	4 (0.8)
**Therapeutic area**		
	Mental health and addictions	111 (21.8)
	Neurology and CNS^a^	78 (15.3)
	Cardiovascular	71 (13.9)
	Metabolism and endocrinology	45 (8.8)
	Oncology	36 (7.1)
	Respiratory and pulmonary	26 (5.1)
	Infectious diseases and vaccines	24 (4.7)
	Rheumatology	22 (4.3)
	Surgery	22 (4.3)
	Pain	14 (2.7)
	Rehabilitation and physical therapy	8 (1.6)
	Musculoskeletal	8 (1.6)
	Urology and nephrology	7 (1.4)
	Gastroenterology	7 (1.4)
	Women’s health	6 (1.2)
	Immunology	6 (1.2)
	Eye health	5 (1)
	Other	5 (1)
	Hematology	4 (0.8)
	Audiology	3 (0.6)
	Dermatology	2 (0.4)
**DHT^b^ types^c^**		
	Smartphone or mobile app	250 (49)
	Wearable	164 (32.2)
	Parameter-specific biosensors	72 (14.1)
	Mobile phone (other)	62 (12.2)
	Assessments via mobile platform	42 (8.2)
	Web-based platform	34 (6.7)
	Implantable	30 (5.9)
	Ingestible	14 (2.7)
	Electronic medication monitors	9 (1.8)
	Environmental sensors	6 (1.2)
	Software	6 (1.2)
	Other	3 (0.6)
**Number of DHTs**		
	1	346 (67.8)
	2	123 (24.1)
	3	31 (6.1)
	4	10 (2)
**Intervention being evaluated**		
	DHT only	352 (69)
	Drug	64 (12.5)
	Program	38 (7.5)
	Procedure	36 (7.1)
	Device (non-DHT)	20 (3.9)
**Data entry type**		
	Passive only	189 (37.1)
	Active only	170 (33.3)
	Hybrid	151 (29.6)
**Type of data collected by DHT^c^**		
	Physiological	189 (37.1)
	Clinical symptoms	188 (36.9)
	Behavioral	171 (33.5)
	Physical activity	165 (32.4)
	Mood	80 (15.7)
	Sleep	59 (11.6)
	Contextual	56 (11)
	Functional	56 (11)
	Cognitive	51 (10)
	Quality of life	41 (8)
	Geospatial	36 (7.1)
	Pain	35 (6.9)
	Demographic	18 (3.5)
	Gait	18 (3.5)
	Experience	14 (2.7)
	Environmental	4 (0.8)
**Analytic applications**		
	Descriptive	380 (74.5)
	Predictive	70 (13.7)
	Diagnostic	33 (6.5)
	Prescriptive	27 (5.3)
**Use of AI^d^ for data collection or measurement**		
	No	449 (88)
	Yes	61 (12)

^a^CNS: central nervous system.

^b^DHT: digital health technology.

^c^Categories are not mutually exclusive.

^d^AI: artificial intelligence.

### Temporal Trends

#### Publications

**Figure 2:**
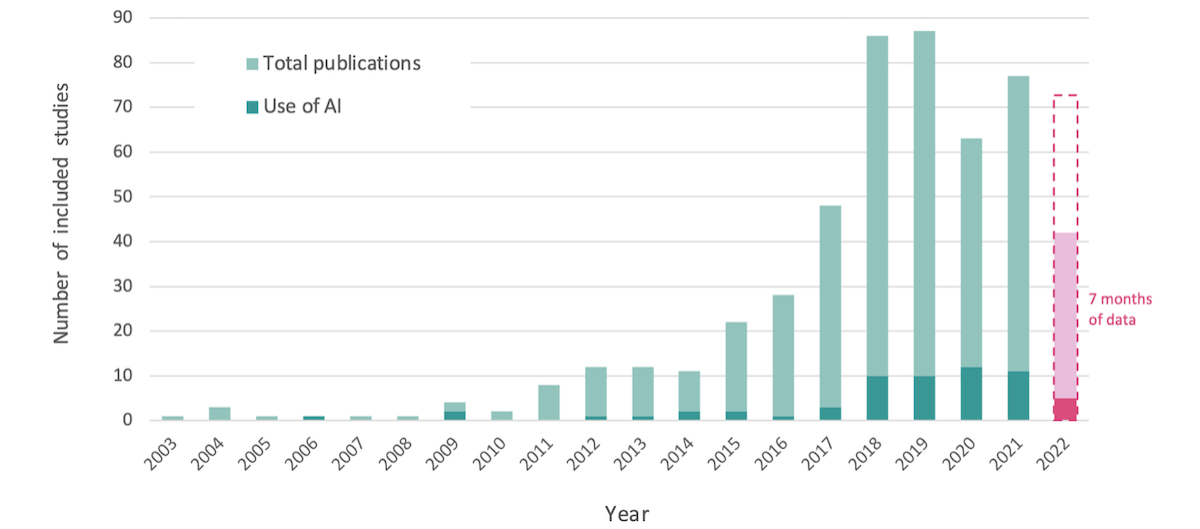
Number of included studies and those involving artificial intelligence (AI) by year.

The number of publications with clinical outcome measurements using DHTs has grown annually since emerging in 2003. Substantial increases occurred between 2015 and 2018, with the greatest number of publications observed in 2019 (87/510, 17.1%). With worldwide scientific publications growing at an average rate of 4% per year, the pre-2018 growth in publications reflects a real increase beyond overall publication trends [24]. A noticeable dip in publications was observed in 2020, but the numbers seemed to be rising again. The incorporation of AI in these studies started to grow in 2018 and has remained consistent in the years since. Given that the last search was run on July 31, 2022, the most recent year’s data reflect only 7 months of publications and should be interpreted in this context, and projections were determined accordingly (Figure 2).

#### DHT Types

**Figure 3:**
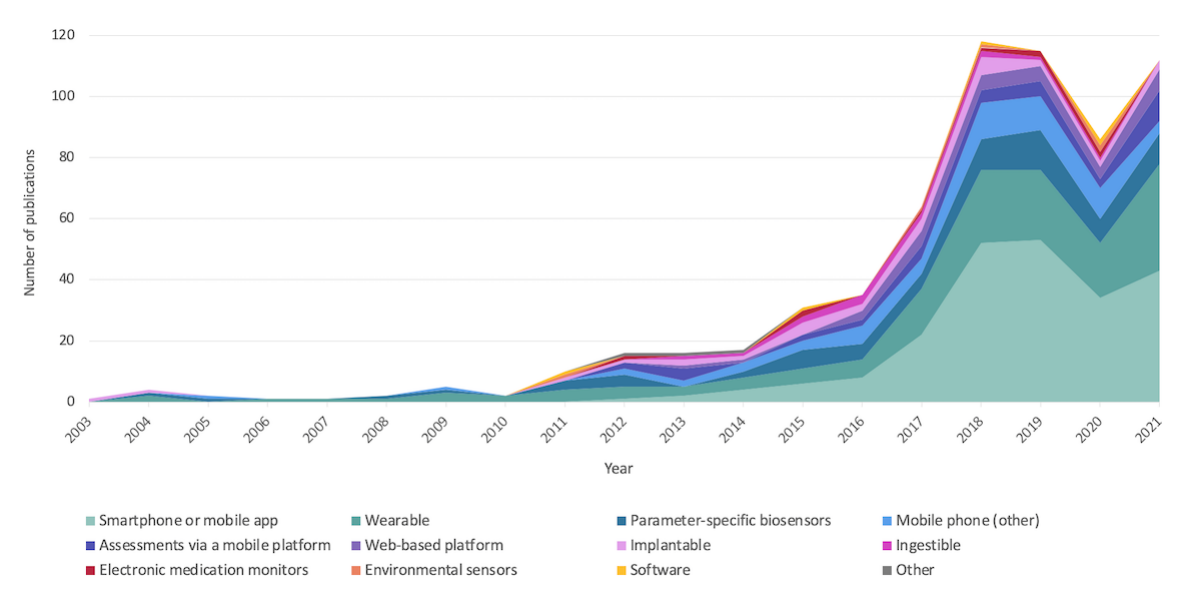
Types of digital health technologies used in the included studies by year.

The use of DHT for real-world clinical outcome measurement started to be captured in publications in 2003, and for the first 7 years, total publications remained relatively low (<5 articles/y). These early years comprised studies on wearables (eg, wrist actigraphy and wearable strain sensor), implantables (eg, implantable loop recorder and implantable glucose sensor), early parameter-specific biosensors (eg, blood pressure monitor), and other nonapp mobile phone approaches. After 2010, we see a growing number of publications and the emergence of studies using environmental sensors and software for outcome measurement, followed shortly thereafter by mobile assessments (eg, ecological momentary assessment), electronic medication monitors, and mobile apps. Since 2012, the use of mobile apps for collecting patient-generated data has grown steadily, and it has been the leading DHT in this literature for the past 6 years. Ingestibles did not appear until 2013; similar to implantables, electronic medication monitors, and environmental biosensors, annual numbers have remained low, and we have yet to see a significant surge in articles using these technologies. Across DHT types, a significant dip in the number of articles was seen in 2020, but since then, most of the DHT types have been on the rise, with wearables and mobile apps continuing to see the greatest growth (Figure 3).

### TAs Overview

**Figure 4:**
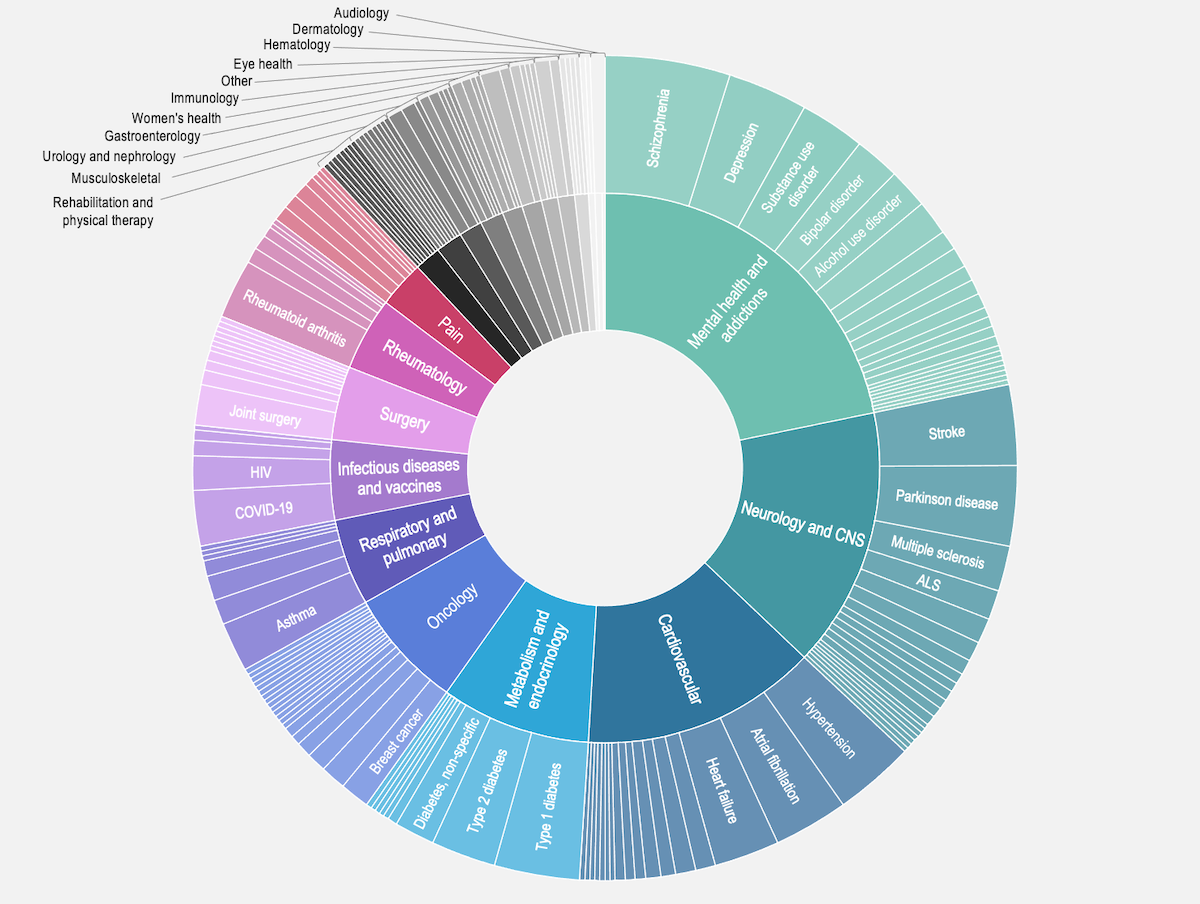
Included studies by therapeutic area and disease or condition. ALS: amyotrophic lateral sclerosis; CNS: central nervous system.

The use of DHT for collecting PGHD and measuring clinical outcomes spans 21 distinct TAs within the included studies. Overall, 51% (n=260) of all 510 studies pertained to 1 of 3 TAs: mental health and addictions (n=111, 21.8%), neurology or CNS (n=78, 15.3%), and cardiovascular (n=71, 13.9%; Figure 4). At the disease or condition level, mental health and addictions is predominantly comprised of studies focused on schizophrenia (25/111, 22.5%), depression (16/111, 14.4%), and opioid or substance use disorder (13/111, 11.7%). For neurology or CNS, stroke (16/78, 21%) and Parkinson disease (16/78, 21%) are the conditions that contributed most significantly; for cardiovascular TA, hypertension (16/71, 23%), atrial fibrillation (15/71, 21%), and heart failure (13/71, 18%) are the largest contributors. The complete list of diseases and conditions that fall within each TA can be found in Table S1 in Multimedia Appendix 4.

### Method of Data Entry

**Figure 5:**
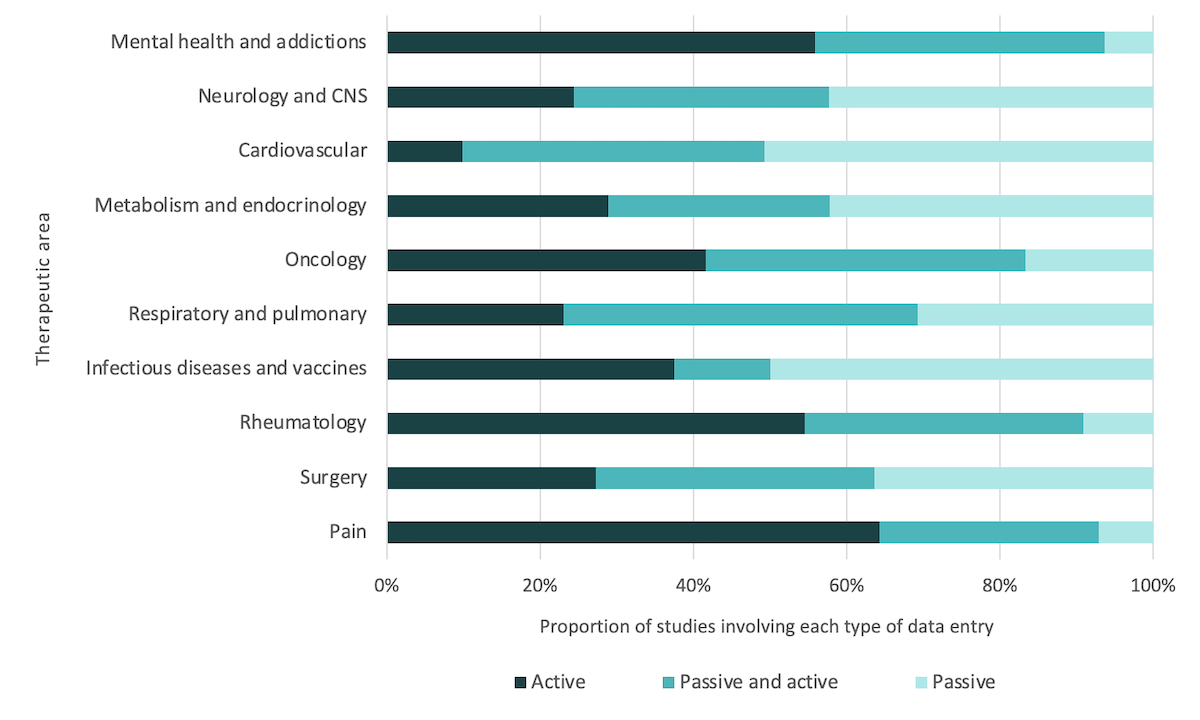
Method of data entry by therapeutic area. CNS: central nervous system.

For each TA, we assessed the proportion of studies that involved active, passive, and hybrid (both active and passive) data collection using DHT. Looking at the 10 most prevalent TAs within this review, all 3 data entry types are represented (Figure 5). The greatest proportion of active data entry was seen within pain (9/14, 64% of studies; eg, patient-reported daily pain severity on a 5-point scale using a smartphone app); mental health and addictions (62/111, 55.9% of studies; eg, ecological momentary assessment of depression symptoms through mobile device prompts to complete the Hamilton rating scale); and rheumatology (12/22, 55% of studies; eg, a smartphone app to track rheumatoid arthritis treatment compliance). Conversely, the highest proportion of passive data entry was observed for cardiovascular (eg, smartphone-based electrocardiographic event recorder) and infectious disease and vaccines (eg, a pill ingestible sensor for HIV medication adherence measurement), both of which saw approximately 50% of studies with passive collection (36 out of 71 studies and 12 out of 24 studies, respectively). Across the full list of TAs, the only areas with no studies involving passive data collection were eye health, audiology, and dermatology.

### Data Collection and Analytical Applications

**Figure 6:**
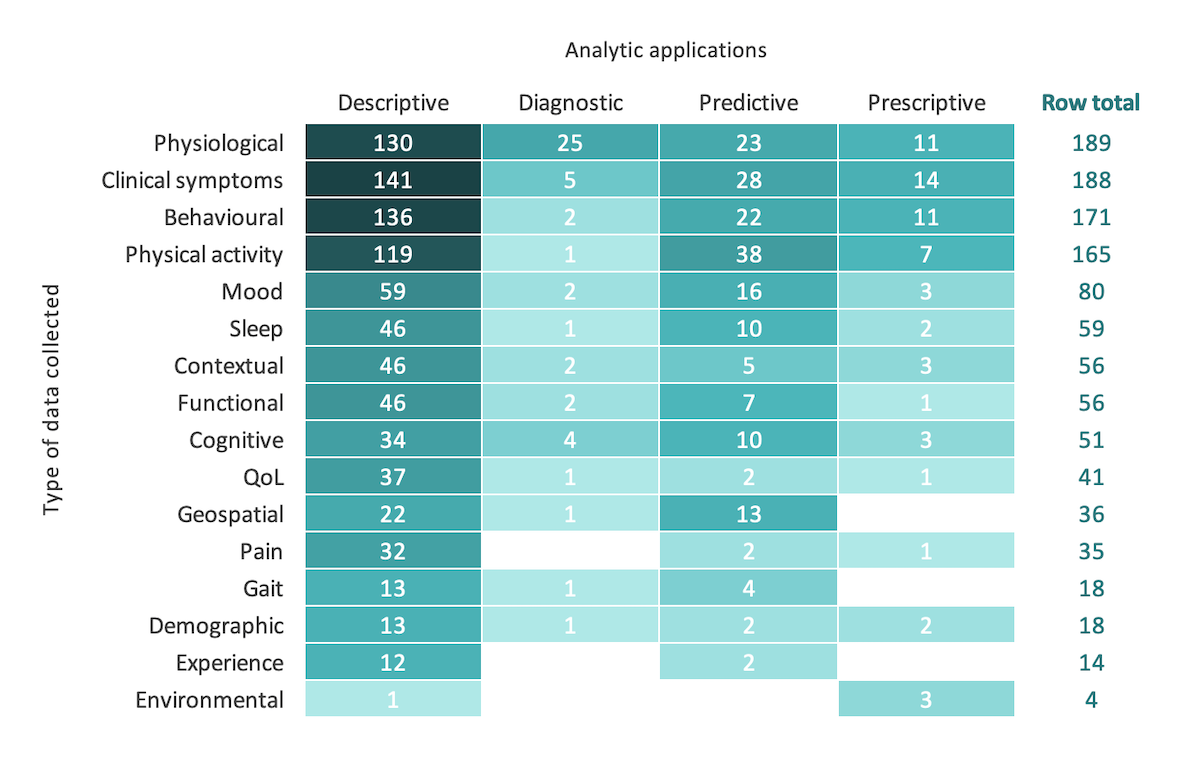
Analytic applications by type of data. QoL: quality of life.

To help visualize the relationship between the different types of data collected by DHTs and their analytical applications, we used a heat map (Figure 6). Data types were classified based on how they were described within each publication, and the final categories appearing in Figure 6 reflect the common groupings that emerged throughout the literature. Descriptive analytics, the predominant application, are seen for studies capturing every type of data—notably, clinical symptoms data (141/380, 37.1%), behavioral data (136/380, 35.8%), physiological data (130/380, 34.2%), and physical activity data (119/380, 31.3%). Diagnostic analytical applications are less common across all studies but consistently appear to align with the collection of physiological data (25/33, 76%; eg, patient-led surveillance of localized melanoma using a mobile dermatoscope). Predictive analytics were most often used in studies collecting physical activity data (38/70, 54%; eg, predicting postdischarge cancer surgery complications using electronic patient-reported outcomes and wearables), with a wide variety of other data types also observed. Relative to other analytical applications, predictive analytics saw the largest proportional contribution for geospatial data (13/70, 19%; eg, monitoring changes in mobility patterns and social behavior for relapse prediction in schizophrenia). Finally, for the least common prescriptive analytical applications, clinical symptoms represent the most common data type (14/27, 52%; eg, the use of multimodal sensors to monitor lung function and tailor asthma care plans). Looking across all types of data, we see that for the more subjective information, such as mood, pain, and experience, there are little to no diagnostic, predictive, or prescriptive applications.

**Figure 7:**
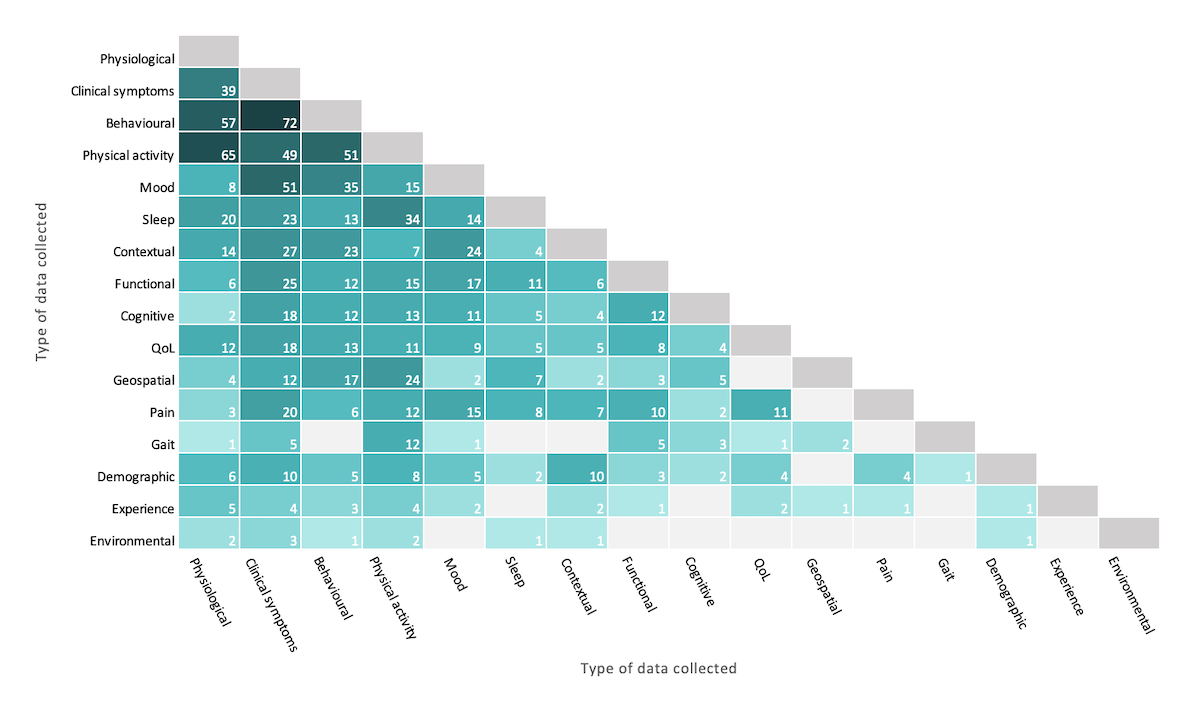
Collection of multiple types of patient-generated data by number of studies. QoL: quality of life.

Most of the studies included in the review involved the collection of more than one type of data (350/510, 68.6%). We explored the interrelationships between the different data types used within the same studies and used a heat map for visualization (Figure 7). The 2 types of data most often collected together were clinical symptoms and behavioral data (72/350, 20.6%; eg, drug craving and drug use), followed by physiological and physical activity data (65/350, 18.6%; eg, heart rate and daily step count). Physiological and behavioral data (57/350, 16.3%), physical activity and behavioral data (51/350, 14.6%), and clinical symptoms and mood data (51/350, 14.6%) were also commonly observed pairs. Physical activity data were also frequently collected with sleep (34/350, 9.7%) and geospatial (24/350, 6.9%) information.

## Discussion

### Principal Findings

#### Overview

This scoping review aimed to systematically explore the use of DHTs for measuring clinical outcomes using patient-generated data. The use of DHTs for RWD collection dates back nearly 20 years, encompassing 169 different diseases and conditions in over 20 TAs and 44 countries. Throughout these years, the timeline for growth in the number of publications is consistent with major milestones in the world of digital health (eg, iPhone and Fitbit launched in 2007 and 2009, respectively) and reflects how commercial interests can drive research [25].

#### DHT-Based Measurement Studies: Through the Years

Since 2018, the growth in the number of studies—and the number of DHT types within those studies—appears to have begun to level out. However, this 2018 to 2021 period was marked by an increase in the use of AI in this body of research. The broad scope of this review is not conducive to drawing a definitive conclusion from this observation—as the need for and successful application of AI are highly dependent on the disease area, use case, and analytical application. With this in mind, our result aligns with the findings that in recent years, select AI tools have progressed past testing to deployment [26], and regulatory approvals for products using AI are occurring at an accelerating rate, particularly for machine learning [27]. Despite advances in AI research and the demonstrated success of AI systems in a variety of retrospective medical studies, a relatively limited number of AI tools have been translated into medical practice [26,28]. In 2020, we observed a significant drop in the number of publications overall and across nearly all types of DHT. This can be expected given the onset of the COVID-19 pandemic and the resulting research interruptions and publishing delays. Interestingly, the total number of studies using AI continued to increase in 2020 despite the emergence of COVID-19. To better understand the impact of the COVID-19 pandemic on digital health measurement—both initially and in the waves of obligatory remote care that followed—further investigation is warranted.

#### TAs of Interest

This study’s cross-TA view also enabled us to compare the use of DHT for RWD collection across TAs. Mental health and addictions represent the TA with the greatest number of studies, comprising 21.8% (111/510) of all included articles. Contributing factors may include within-disease heterogeneity of clinical presentation and a lack of validated biochemical markers to inform diagnosis and prognosis [29]. The latter refers to the fact that, for many mental illnesses, we cannot rely on a definitive laboratory test or biometric to characterize a patient’s current health status. Rather, there is a reliance on patient reporting and clinician observation of symptoms. Other key mental health measures—behavior, cognition, and mood—are rarely captured by clinicians [30]. Where symptoms or other measures are captured in real-world clinical practice, much of these data exist buried in the unstructured, narrative text of the patient’s medical record. Accordingly, DHTs help to fill some of these RWD gaps through their proximity to patients in their real-world environments, where critical mental health outcomes of interest can be collected unobtrusively. Our results showed that for mental health and addictions, most of the DHT data are collected actively (62/111, 55.9%) or through hybrid (passive and active) collection (42/111, 37.8%); however, we expect that as technologies continue to evolve, an increasing number of passive data-collection approaches will be studied and validated.

Digital health capabilities are widely used in neurology research. Within this TA, we saw a greater spread across passive, active, and hybrid data-collection approaches and a wide variety in the types of data and outcomes collected. For instance, both acute conditions, such as ischemic stroke, and progressive neurodegenerative conditions, such as Parkinson disease, necessitate diverse data inputs to measure motor, cognitive, and functional impairment for a given patient. It makes sense that for the included studies on neurological conditions, we observed an array of DHT types and outcome measures involving physical activity, gait, and geolocation. DHTs that collect data actively and passively in a single device represent a promising area for scientific research. A common example we saw in this review for neurology and other TAs was Beiwe [31], an application developed specifically for use in smartphone-based digital phenotyping research that can collect active survey data as well as passive phone sensor data (eg, GPS) and phone use logs.

Other TAs with a substantial body of evidence on DHT-measured outcomes, which may be driven by well-developed technologies with the ability to collect key physiological data, including metabolism or endocrinology and cardiovascular data. The former is predominantly comprised of type 1 and type 2 diabetes, for which blood glucose measurement and disease management have been revolutionized by continuous glucose monitors. For cardiovascular TA, blood pressure and heart rate are the outcomes of interest across several conditions spanning hypertension, atrial fibrillation, and heart failure. Blood pressure has proven to be widely captured by wearables and estimated by smartphone sensors, often in conjunction with other outcomes, and spanning studies within and outside of cardiovascular research. Heart rate and heart rhythm are measurable by a variety of digital devices, and in recent years, this has included more advanced technologies, such as a mobile electrocardiographic recorder (eg, AliveCor Kardia) and disposable multisensor patch (eg, VitalConnect).

#### Data Types Collected From DHTs

The extraction output from this review (Multimedia Appendix 5 [9,32,33]) provides a view of hundreds of unique clinical outcomes measured using DHTs. For analysis and reporting purposes, these outcomes were categorized into 16 distinct types of data. It is not surprising to see physiological, clinical symptoms, behavioral, and physical activity data as the most common, given our understanding of (1) their relevance across conditions, (2) the availability of DHTs capable of collecting these types of data, and (3) their traditional acceptance by health care system stakeholders. In the coming years, there may be an increase in DHT studies capturing more subjective but patient-relevant information, such as mood, quality of life, pain, and patient experience.

#### Sociomarkers

Despite the growing recognition of the social determinants of health, there have been limited applications of social factors for clinical decision-making and DHT research. We found that only 11% (56/510) of all studies included contextual data, 7.1% (36/510) included geospatial data, 3.5% (18/510) included demographic data, and 0.8% (4/510) included environmental data. Where people live, what they do throughout the day, and their sociodemographic status can affect a person’s health and well-being. Recognizing the impact of social factors on health, the concept of “sociomarkers,” indicators of social conditions in which a patient is embedded, has been introduced [34]. Methodological advancements in AI and the use of machine learning–based classification models can enable enhanced disease detection and clinical decision-making using sociomarkers and other data. For instance, Shin et al [34] proved that sociomarkers (eg, poverty level, blight prevalence, and housing quality) can predict health outcomes at the individual level in pediatric asthma cases with 61% accuracy. Future studies, when deliberating what outcomes should be measured by DHTs, should consider how the collection (actively or passively) of these data types may complement the primary outcomes of the study.

#### Digital Biomarkers

Digital biomarkers serve as an indicator of biological processes or responses, can cover a broad range of measurements. Susceptibility or risk, diagnostic, monitoring, prognostic, predictive, and pharmacodynamic or response biomarkers have all been cited as categories for the classification of both digital and traditional biomarkers [35]. A distinct but related classification exists for types of health analytics: descriptive, diagnostic, predictive, and prescriptive [36]. What is evident across both sets of categories is that more advanced predictive and prescriptive applications necessitate a richness of data—including but not limited to depth and breadth of information, comprehensive historical data, hybrid data collection, multimodal data, and composite measures—to answer the research question of interest [37]. This notion aligns with our findings that studies that go beyond descriptive analytical applications are in the minority. However, the research that does exist represents an exciting and evolving space where objective, ecological measures have the ability to transform clinical practice. This is particularly true for neuropsychiatry and neurodegeneration, from the detection of age-related changes in bipolar disorder using a passive smartphone kinematics-based digital biomarker [32] to the prediction of onset of falls, cognitive impairment, and functional impairment in Parkinson disease using a simple smartphone test [33].

The collection of more data over more time points, the integration of different data types, and the combination of digital biomarkers can enable the development of phenotypic signatures to improve the understanding and monitoring of disease states [38]. This scoping review demonstrates the volume, variety, and velocity of “big data” that can be collected by DHTs and the contribution of these data to bolstering disease phenotypes. As digital technologies continue to advance alongside analytical capabilities, we can expect meaningful progress toward personalized health and precision medicine.

### Strengths and Limitations

This is the first study, to our knowledge, to systematically review the real-world outcomes of DHTs across all geographical and disease areas. The systematic nature of this scoping review underpinned the research process end to end, from early design and registration of the protocol to the thorough screening and eligibility criteria. The resulting comprehensive repository of studies provides a depth and breadth of data in a usable format for researchers, regulators, and the industry alike. This scoping review has several limitations. As described in the *Methods* section, multiple reviewers were used to pilot the inclusion and exclusion criteria and review an initial subset of abstracts; however, most reviews were performed by a single reviewer and may have been prone to selection bias and human error. Another limitation is the use of only 1 database in the updated search in 2022. This was a strategic decision to select the database from which the vast majority of the initially included studies from the 2021 search were derived and conduct a more efficient search. Moreover, the decision to exclude studies that solely involved step measurements (ie, by pedometer or accelerometer) means that our results underrepresent the total number of articles capturing physical activity data and passive-only measurements. Although we did include studies where physical activity is measured with other data types, we recognized that there is a subset of wearables and mobile apps that we may be excluding if only steps are collected. A limitation of this study also relates to the absence of an established classification framework for DHT types and applications, prompting the development of the key terms and concepts in Table 1. Given that a single study may involve more than one of the DHT types and a single DHT may collect multiple types of data, interpretation of the results must consider that these labels are not mutually exclusive. Finally, the studies included in this review were largely conducted in high-income countries in North America and Western Europe, as is reflective of most published research. It is beyond the scope of this study to assess what else, if anything, may be driving this trend, but given most of the world has access to digital technology today and data-driven approaches are used to understand patients across the world, it is worth considering whether our detailed eligibility criteria may contribute to this finding. For example, the exclusion of studies that rely on clinical site or provider involvement may disproportionately exclude low- and middle-income countries where current DHTs are not yet fully autonomous and remote.

### Implications for Future Research

Further work is needed to investigate the impact of the included studies on particular areas of research. In mental health research, where we are seeing momentum in the use of digital biomarkers, further systematic review and synthesis would be useful to understand the nature and quality of evidence on DHTs for characterizing patient experience and predicting critical outcomes such as relapse. With many studies centered around the detection and monitoring of COVID-19 and peak international interest in pandemic preparedness, research could leverage DHTs to more broadly measure and predict the risk of disease transmission during disease outbreaks. DHT research as a whole demands more rigorous and reproducible research designs, larger sample sizes, longer follow-up times, and greater consistency in the metrics used.

Title and abstract screening revealed that most research in this space occurs in “non–real-world” settings. These excluded articles largely represent studies involving the testing and evaluation of digital technologies in highly controlled, often laboratory-based settings. Before a DHT is implemented in clinical practice or used by patients and consumers in their day-to-day life, there are several stages of technology verification (evaluation of sensor performance), analytical validation (evaluation of algorithm performance), and clinical validation (evaluation of the ability to measure outcomes within the context of interest) [39]. Additional research could help identify the main barriers to technologies proceeding past early testing and validation and toward real-world studies.

The evaluation of novel DHTs may indeed yield negative results before the technology reaches real-world patients, but beyond performance shortcomings, researchers should also consider the influence of patient-important factors such as usability and acceptability. Even the most accurate and reliable tools may see limited adoption and minimal clinical utility if the patient experience is negative. With the patient perspective in mind, another key consideration is whether these DHTs used for outcome measurement are widening or bridging the digital divide—the gap in access to and use of technology between different sociodemographic groups and regions [40]. For example, a study providing DHTs to participants may demonstrate positive real-world outcomes and experiences; however, if the DHT cost is prohibitive outside of the study setting, then certain populations will be disproportionately excluded from its benefits. More targeted systemic reviews examining select DHTs or outcomes should consider assessing the literature for factors contributing to the digital divide, including lack of access to the internet or smartphones, low literacy or digital literacy, and distrust in technology or the people who govern it.

### Conclusions

This research serves to systematically map and summarize the existing evidence on the use of DHTs for measuring clinical outcomes using patient-generated data. The 510 articles included in this scoping review offer a comprehensive view of the variety of types of technology, data, collection methods, analytical approaches, and therapeutic applications within this growing body of evidence. The presented results and repository of studies can help to identify gaps and opportunities to inform future research, including subsequent systematic reviews. Through their remote capabilities and proximity to patients, there is potential for DHTs to unlock RWD that can be used to diagnose disease, characterize health status, and predict health outcomes.

## Appendix

Multimedia Appendix 1 PRISMA-ScR (Preferred Reporting Items for Systematic Reviews and Meta-Analyses extension for Scoping Reviews) checklist.

Multimedia Appendix 2 Inclusion and exclusion criteria.

Multimedia Appendix 3 Detailed search strategy.

Multimedia Appendix 4 Geographical distribution of included studies by country of origin and summary of studies by disease and therapeutic area.

Multimedia Appendix 5 Full data extraction.

## Abbreviations

AI: artificial intelligence
CNS: central nervous system
DHT: digital health technology
PGHD: patient-generated health data
PRISMA-ScR: Preferred Reporting Items for Systematic Reviews and Meta-Analyses extension for Scoping Reviews
RWD: real-world data
TA: therapeutic area

## Data Availability

All data generated or analyzed during this study are included in this paper (Multimedia Appendix 5).
