# Supplementary material for: Digital Health Technology for Real-World Clinical Outcome Measurement Using Patient-Generated Data: Systematic Scoping Review
Source: J Med Internet Res. 2023 Oct 11;25:e46992. doi: 10.2196/46992 (PMC10600647; doi:10.2196/46992)
Supplement: Multimedia Appendix 2 [file jmir_v25i1e46992_app2.docx]

## Inclusion and exclusion criteria

| **Criterion** | **Inclusion Criteria** | **Exclusion Criteria** |
| --- | --- | --- |
| **Sources / Study Types** | - Primary studies, both experimental and observational (cohort, case-control) studies will be considered.   *This includes:*   - Experimental and quasi-experimental study designs (e.g., randomized controlled trials, non-randomized controlled trials, and before and after studies) - Analytical observational studies (e.g., prospective and retrospective cohort studies, case-control studies and analytical cross-sectional studies) - Descriptive observational study designs (e.g., case series, individual case reports and descriptive cross-sectional studies) - Studies that focus on quantitative data will be considered - Studies focused on the *applications* of digital technologies (incl. pilot studies and clinical feasibility studies) will be considered | - Purely qualitative studies - All reviews - All commentaries, editorials - Studies without original data - Studies focused on technology validation (e.g., sensor accuracy or software performance), algorithm development, and/or operational feasibility |
| **Participants** | - All persons/patients receiving a clinical intervention, irrespective of age, sex, gender, diagnoses, comorbidities, symptom severity or treatment stage/setting will be considered. | - No defined participant population - Therapeutic area or health condition not clearly stated |
| **Concept:** DHT | - Studies involve digital health technologies (DHT) for the collection of data on an accompanying clinical intervention or for measuring any clinical outcome/endpoint. - Data being collected (i) must be exclusively “patient-generated”, and (ii) must include health-related data (e.g., biometrics, digital biomarkers, clinical outcomes). - DHT must be connected (to the internet or via Bluetooth, mobile app or USB), and it must not require any clinician involvement for the patient to generate data in their day-to-day life. | - DHT is only used as an intervention - DHT is used exclusively for study recruitment and retention - DHT requires clinician involvement to collect data |
| **Concept:** Clinical Intervention | - Any “clinical”/ “medical”/ “therapeutic” intervention, such as a drug treatment, surgical procedure, diagnostic test or psychological therapy can be included as the accompanying clinical intervention being measured by the DHT. | - Interventions that are not focused on individual-level care. Examples of such exclusions are: public health interventions, encompassing population-wide health promotion and disease prevention efforts (e.g. healthy diet and physical activity); and social care interventions, such as support for primary caregivers. |
| **Concept:** Outcomes | - Any clinical endpoint/outcome, digital biomarker, or predictor of clinical outcomes or intervention effectiveness. This includes measures of health status, disease progression, functioning, quality of life, mental health, safety/adverse events, predictors of treatment response or failure. | - No clinical outcome identified. |
| **Context:** | - Studies in which digital health data are collected from patients (either automatic/passive or manual/active entry) in their day-to-day life outside of clinical visits. | - Studies in which the data collection context is reliant on live interactions with healthcare providers (e.g., primary care physician) or institutions (e.g., clinic, hospital) |
| **Other** | - Studies published since January 1, 2000 - Studies published in English | - Studies published before January 1, 2000 - Studies not available in English |
