# Supplementary material for: Digital Health Technology for Real-World Clinical Outcome Measurement Using Patient-Generated Data: Systematic Scoping Review
Source: J Med Internet Res. 2023 Oct 11;25:e46992. doi: 10.2196/46992 (PMC10600647; doi:10.2196/46992)
Supplement: Multimedia Appendix 3 [file jmir_v25i1e46992_app3.docx]

## Scoping Review Search Strategy

MEDLINE (Ovid) Search

| **Concept** | **Layer** | **Search Terms** |
| --- | --- | --- |
| Digital Health Technology | 1 | Wireless Technology/ |
|  | 2 | Mobile Applications/ |
|  | 3 | wearable electronic devices/ or fitness trackers/ |
|  | 4 | Smartphone/ |
|  | 5 | (digital adj2 (tool? or device?)).ti,ab. |
|  | 6 | (wearable* adj2 (device? or sensor? or motion*)).ti,ab. |
|  | 7 | (wearable* adj2 (technolog* or monitor* or track*)).ti,ab. |
|  | 8 | (pedometer? or accelerometer?).ti,ab. |
|  | 9 | (mobile adj2 (technolog* or device?)).ti,ab. |
|  | 10 | (inertial adj2 (sensor? or device?)).ti,ab. |
|  | 11 | (ingestible adj2 (device? or technolog*)).ti,ab. |
|  | 12 | (activity adj2 (track* or monitor*)).ti,ab. |
|  | 13 | (smartphone? or (smart adj2 (phone? or device?))).ti,ab. |
|  | 14 | (Fitbit? or Apple watch* or Smartwatch* or ResearchKit? or HealthKit? or Healthpatch* or Garmen? or patient-facing technolog* or GPS system? or Global Positioning System? or mHealth technolog* or Textile piezoresistive sensor? or smartphone? or biochip? or digital implant? or implant technolog* or portable software or portable electronic app* or fitness track* or embedded sensor? or sensor technolog* or remote technolog* or wireless sensor? or motion track*).ti,ab. |
|  | 15 | 1 or 2 or 3 or 4 or 5 or 6 or 7 or 8 or 9 or 10 or 11 or 12 or 13 or 14 |
| Digital Health Data | 16 | exp Monitoring, Physiologic/ |
|  | 17 | (patient adj (monitor* or track* or generated*)).ti,ab. |
|  | 18 | (real world adj2 (evidence or data)).ti,ab. |
|  | 19 | (outcome? adj2 (measure* or predict*)).ti,ab. |
|  | 20 | (digital biomarker? or digital phenotyp* or ecological momentary assessment?).ti,ab. |
|  | 21 | 16 or 17 or 18 or 19 or 20 |
| Outcome Measurement | 22 | Outcome Assessment, Health Care/ |
|  | 23 | exp patient outcome assessment/ |
|  | 24 | exp patient reported outcome measures/ |
|  | 25 | exp "Outcome and Process Assessment, Health Care"/ |
|  | 26 | exp treatment outcome/ |
|  | 27 | "Recovery of Function"/ |
|  | 28 | patient admission/ or patient discharge/ or patient readmission/ |
|  | 29 | patient compliance/ or medication adherence/ |
|  | 30 | "Quality of Life"/ |
|  | 31 | exp Mortality/ |
|  | 32 | "Activities of Daily Living"/ |
|  | 33 | exp "Treatment Adherence and Compliance"/ |
|  | 34 | ((clinical or treatment or patient? or surrogate) adj3 (outcome* or endpoint*)).ti,ab. |
|  | 35 | ((treatment* or drug* or medication* or clinical) adj2 (adherence or nonadherence or compliance or noncompliance or effectiveness or efficacy or failure? or response* or surveillance)).ti,ab. |
|  | 36 | ((adverse or safety or unintended or unintentional) adj2 (interaction? or response? or effect? or event? or reaction? or outcome?)).ti,ab. |
|  | 37 | (emergency adj2 (visit* or stay)).ti,ab. |
|  | 38 | (patient experience? or patient impact? or "length of stay" or "length of hospital stay" or complication? or health resource utili#ation or healthcare utili#?ation or hospitali#ation? or admission or readmission? or re-admission? or ED visit? or ER visit? or recovery or "quality of life" or QoL or "activities of daily living" or ADL or health status or disease progression or disease control or function* or survival or mental health outcome? or relapse* or symptomatic response).ti,ab. |
|  | 39 | 22 or 23 or 24 or 25 or 26 or 27 or 28 or 29 or 30 or 31 or 32 or 33 or 34 or 35 or 36 or 37 or 38 |
|  | 40 | 15 and 21 and 39 |
| Study Type | 41 | (comment or editorial or letter).pt. |
|  | 42 | 40 not 41 |
| Other | 43 | 42 not (exp animals/ NOT humans/) |
|  | 44 | limit 43 to (english language and yr="2000 -Current") |

CINAHL Search

| **Concept** | **Layer** | **Search Terms** |
| --- | --- | --- |
| Digital Health Technology | S1 | (MH "Mobile Applications") |
|  | S2 | (MH "Accelerometers") OR (MH "Biophysical Instruments") OR (MH "Wearable Sensors") OR (MH "Smart Glasses") OR (MH "Electrodes, Implanted") OR (MH "Fitness Trackers") OR (MH "Pedometers") |
|  | S3 | (MH "Smartphone") |
|  | S4 | TI (digital N1 (tool? OR device?)) OR AB (digital N1 (tool? OR device?)) |
|  | S5 | TI (wearable* N1 (device? OR sensor? OR motion*)) OR AB (wearable* N1 (device? OR sensor? OR motion*)) |
|  | S6 | TI (wearable* N1 (technolog* OR monitor* OR track*)) OR AB (wearable* N1 (technolog* OR monitor* OR track*)) |
|  | S7 | TI (pedometer? OR accelerometer?)) OR AB (pedometer? OR accelerometer?)) |
|  | S8 | TI (mobile N1 (technolog* OR device?)) OR AB (mobile N1 (technolog* OR device?)) |
|  | S9 | TI (inertial N1 (sensor? OR device?)) OR AB (inertial N1 (sensor? OR device?)) |
|  | S10 | TI (ingestible N1 (device? OR technolog*)) OR AB (ingestible N1 (device? OR technolog*)) |
|  | S11 | TI (activity N1 (track* OR monitor*)) OR AB (activity N1 (track* OR monitor*)) |
|  | S12 | TI (smart N1 (phone? OR device?)) OR AB (smart N1 (phone? OR device?)) |
|  | S13 | TI (Fitbit? OR “Apple watch*” OR Smartwatch* OR ResearchKit? OR HealthKit? OR Healthpatch* OR Garmen? OR “patient-facing technolog*” OR “GPS system?” OR “Global Positioning System” OR “mHealth technolog*” OR “Textile piezoresistive sensor?” OR smartphone? OR biochip? OR “digital implant?” OR “implant technolog*” OR “portable software” OR “portable electronic app*” OR “fitness track*” OR “embedded sensor?” OR “sensor technolog*” OR “remote technolog*” OR “wireless sensor?” OR “motion track*”) OR AB (Fitbit? OR “Apple watch*” OR Smartwatch* OR ResearchKit? OR HealthKit? OR Healthpatch* OR Garmen? OR “patient-facing technolog*” OR “GPS system?” OR “Global Positioning System” OR “mHealth technolog*” OR “Textile piezoresistive sensor?” OR smartphone? OR biochip? OR “digital implant?” OR “implant technolog*” OR “portable software” OR “portable electronic app*” OR “fitness track*” OR “embedded sensor?” OR “sensor technolog*” OR “remote technolog*” OR “wireless sensor?” OR “motion track*”) |
|  | S14 | S1 OR S2 OR S3 OR S4 OR S5 OR S6 OR S7 OR S8 OR S9 OR S10 OR S11 OR S12 OR S13 |
| Digital Health Data | S15 | (MH "Monitoring, Physiologic+") |
|  | S16 | TI (patient N0 (monitor* OR track* OR generated*)) OR AB (patient N0 (monitor* OR track* OR generated*)) |
|  | S17 | TI (“real world” N1 (evidence OR data)) OR AB (real-world N1 (evidence OR data)) |
|  | S18 | TI (outcome? N1 (measure* OR predict*)) OR AB (outcome? N1 (measure* OR predict*)) |
|  | S19 | TI (“digital biomarker?” OR “digital phenotyp*” OR “ecological momentary assessment?”) OR AB (“digital biomarker?” OR “digital phenotyp*” OR “ecological momentary assessment?”) |
|  | S20 | S15 OR S16 OR S17 OR S18 OR S19 |
| Outcome Measurement | S21 | (MH "Outcome Assessment") |
|  | S22 | (MH "Patient-Reported Outcomes+") |
|  | S23 | (MH "Outcomes (Health Care)") |
|  | S24 | (MH "Process Assessment (Health Care)") |
|  | S25 | (MH "Treatment Outcomes+") OR (MH "Drug Efficacy") OR (MH "Therapeutic Index") OR (MH "Treatment Failure") OR (MH "Treatment Termination") |
|  | S26 | (MH "Functional Status") |
|  | S27 | (MH "Hospitalization") OR (MH "Patient Admission") OR (MH "Patient Discharge") OR (MH "Readmission") |
|  | S28 | (MH "Patient Compliance") OR (MH "Medication Compliance") |
|  | S29 | (MH "Quality of Life") |
|  | S30 | (MH "Mortality+") |
|  | S31 | (MH "Activities of Daily Living") |
|  | S32 | TI ((clinical OR treatment OR patient? OR surrogate) N2 (outcome* OR endpoint*)) OR AB ((clinical OR treatment OR patient? OR surrogate) N2 (outcome* OR endpoint*)) |
|  | S33 | TI ((treatment* OR drug* OR medication* OR clinical) N1 (adherence OR nonadherence OR compliance OR noncompliance OR effectiveness OR efficacy OR failure? OR response* OR surveillance)) OR AB ((treatment* OR drug* OR medication* OR clinical) N1 (adherence OR nonadherence OR compliance OR noncompliance OR effectiveness OR efficacy OR failure? OR response* OR surveillance)) |
|  | S34 | TI ((adverse or safety or unintended or unintentional) N1 (interaction* or response* or effect* or event* or reaction* or outcome*)) OR AB ((adverse or safety or unintended or unintentional) N1 (interaction* or response* or effect* or event* or reaction* or outcome*)) |
|  | S35 | TI (emergency N1 (visit* OR stay)) OR AB (emergency N1 (visit* OR stay)) |
|  | S36 | TI (“patient experience?” or “patient impact?” or “length of stay” or “length of hospital stay” or complication? or “health resource utili?ation” or “healthcare utili?ation” or hospitali?ation? or admission or readmission? or re-admission? or “ED visit?” or “ER visit?” or recovery or “quality of life” or QoL or “activities of daily living” or ADL or “health status” or “disease progression” or “disease control” or function* or survival or “mental health outcome?” or relapse* or “symptomatic response?”) OR AB (“patient experience?” or “patient impact?” or “length of stay” or “length of hospital stay” or complication? or “health resource utili?ation” or “healthcare utili?ation” or hospitali?ation? or admission or readmission? or re-admission? or “ED visit?” or “ER visit?” or recovery or “quality of life” or QoL or “activities of daily living” or ADL or “health status” or “disease progression” or “disease control” or function* or survival or “mental health outcome?” or relapse* or “symptomatic response?”) |
|  | S37 | S21 OR S22 OR S23 OR S24 OR S25 OR S26 OR S27 OR S28 OR S29 OR S30 OR S31 OR S32 OR S33 OR S34 OR S35 OR S36 |
|  | S38 | S14 AND S20 AND S37 |
| Study Type | S39 | PT (Editorial OR Letter OR Commentary) |
|  | S40 | S38 NOT S39 |
| Other | S41 | S40 NOT (((MH "Animals+") OR (MH "Animal Studies") OR (TI "animal model*")) NOT (MH "human")) |
|  | S42 | Limiters - Published Date: 20000101-20211231  Narrow by Language: - english |

Cochrane (CENTRAL) Search

| **Concept** | **Layer** | **Search Terms** |
| --- | --- | --- |
| Digital Health Technology | #1 | [mh ^“Wireless Technology”] |
|  | #2 | [mh ^“Mobile Applications”] |
|  | #3 | [mh ^“wearable electronic devices”] OR [mh ^“fitness trackers”] |
|  | #4 | [mh ^“Smartphone”] |
|  | #5 | (digital NEAR/2 (tool? OR device?)):ti,ab |
|  | #6 | (wearable* NEAR/2 (device* OR sensor* OR motion*)):ti,ab |
|  | #7 | (wearable* NEAR/2 (technolog* OR monitor* OR track*)):ti,ab |
|  | #8 | (pedometer* OR accelerometer*):ti,ab |
|  | #9 | (mobile NEAR/2 (technolog* OR device*)):ti,ab |
|  | #10 | (inertial NEAR/2 (sensor* OR device*)):ti,ab |
|  | #11 | (ingestible NEAR/2 (device* OR technolog*)):ti,ab |
|  | #12 | (activity NEAR/2 (track* OR monitor*)):ti,ab |
|  | #13 | (smart NEAR/2 (phone* OR device*)):ti,ab |
|  | #14 | (Fitbit* OR “Apple watch*” OR Smartwatch* OR ResearchKit* OR HealthKit* OR Healthpatch* OR Garmen* OR “patient-facing technolog*” OR “GPS system*” OR “Global Positioning System” OR “mHealth technolog*” OR “Textile piezoresistive sensor*” OR smartphone* OR biochip* OR “digital implant*” OR “implant technolog*” OR “portable software” OR “portable electronic app*” OR “fitness track*” OR “embedded sensor*” OR “sensor technolog*” OR “remote technolog*” OR “wireless sensor*” OR “motion track*”):ti,ab |
|  | #15 | #1 OR #2 OR #3 OR #4 OR #5 OR #6 OR #7 OR #8 OR #9 OR #10 OR #11 OR #12 OR #13 OR #14 |
| Digital Health Data | #16 | [mh “Monitoring, Physiologic”] |
|  | #17 | (patient NEAR/1 (monitor* OR track* OR generated*)):ti,ab |
|  | #18 | (“real world” NEAR/2 (evidence OR data)):ti,ab |
|  | #19 | (outcome? NEAR/2 (measure* OR predict*)):ti,ab |
|  | #20 | (“digital biomarker*” OR “digital phenotyp*” OR “ecological momentary assessment*”):ti,ab |
|  | #21 | #16 OR #17 OR #18 OR #19 OR #20 |
| Outcome Measurement | #22 | [mh ^“Outcome Assessment, Health Care”] |
|  | #23 | [mh “patient outcome assessment”] |
|  | #24 | [mh "patient reported outcome measures”] |
|  | #25 | [mh "Outcome and Process Assessment, Health Care"] |
|  | #26 | [mh “treatment outcome”] |
|  | #27 | [mh ^"Recovery of Function"] |
|  | #28 | [mh ^“patient admission”] OR [mh ^“patient discharge”] OR [mh ^“patient readmission”] |
|  | #29 | [mh ^“patient compliance”] OR [mh ^“medication adherence”] |
|  | #30 | [mh ^"Quality of Life"] |
|  | #31 | [mh "Mortality”] |
|  | #32 | [mh ^"Activities of Daily Living"] |
|  | #33 | [mh "Treatment Adherence and Compliance"] |
|  | #34 | ((clinical OR treatment OR patient* OR surrogate) NEAR/3 (outcome* OR endpoint*)):ti,ab |
|  | #35 | ((treatment* OR drug* OR medication* OR clinical) NEAR/2 (adherence OR nonadherence OR compliance OR noncompliance OR effectiveness OR efficacy OR failure* OR response* OR surveillance)):ti,ab |
|  | #36 | ((adverse or safety or unintended or unintentional) NEAR/2 (interaction* or response* or effect* or event* or reaction* or outcome*)):ti,ab |
|  | #37 | (emergency NEAR/2 (visit* OR stay)):ti,ab |
|  | #38 | (“patient experience*” OR “patient impact*” OR “length of stay” OR “length of hospital stay” OR complication* OR “health resource utili?ation” OR “healthcare utili?ation” OR hospitali?ation* OR admission OR readmission* OR re-admission OR ED visit* OR ER visit* OR recovery OR “quality of life” OR QoL OR “activities of daily living” OR ADL OR “health status” OR “disease progression” OR “disease control” OR function* OR survival OR “mental health outcome*” OR relapse* OR “symptomatic response*”):ti,ab |
|  | #39 | #22 OR #23 OR #24 OR #25 OR #26 OR #27 OR #28 OR #29 OR #30 OR #31 OR #32 OR #33 OR #34 OR #35 OR #36 OR #37 OR #38 |
|  | #40 | #15 AND #21 AND #39 |
| Other | #41 | Limit: with Publication Year from 2000 to 2021 |

Embase Search

| **Concept** | **Layer** | **Search Terms** |
| --- | --- | --- |
| Digital Health Technology | 1 | wireless communication/ |
|  | 2 | exp mobile application/ |
|  | 3 | wearable computer/ or smart watch/ or activity tracker/ |
|  | 4 | smartphone/ |
|  | 5 | (digital adj2 (tool? or device?)).ti,ab. |
|  | 6 | (wearable* adj2 (device? or sensor? or motion*)).ti,ab. |
|  | 7 | (wearable* adj2 (technolog* or monitor* or track*)).ti,ab. |
|  | 8 | (pedometer? or accelerometer?).ti,ab. |
|  | 9 | (mobile adj2 (technolog* or device?)).ti,ab. |
|  | 10 | (inertial adj2 (sensor? or device?)).ti,ab. |
|  | 11 | (ingestible adj2 (device? or technolog*)).ti,ab. |
|  | 12 | (activity adj2 (track* or monitor*)).ti,ab. |
|  | 13 | (smartphone? or (smart adj2 (phone? or device?))).ti,ab. |
|  | 14 | (Fitbit? or Apple watch* or Smartwatch* or ResearchKit? or HealthKit? or Healthpatch* or Garmen? or patient-facing technolog* or GPS system? or Global Positioning System? or mHealth technolog* or Textile piezoresistive sensor? or smartphone? or biochip? or digital implant? or implant technolog* or portable software or portable electronic app* or fitness track* or embedded sensor? or sensor technolog* or remote technolog* or wireless sensor? or motion track*).ti,ab. |
|  | 15 | 1 or 2 or 3 or 4 or 5 or 6 or 7 or 8 or 9 or 10 or 11 or 12 or 13 or 14 |
| Digital Health Data | 16 | physiologic monitoring/ |
|  | 17 | (patient adj (monitor* or track* or generated*)).ti,ab. |
|  | 18 | (real world adj2 (evidence or data)).ti,ab. |
|  | 19 | (outcome? adj2 (measure* or predict*)).ti,ab. |
|  | 20 | (digital biomarker? or digital phenotyp* or ecological momentary assessment?).ti,ab. |
|  | 21 | 16 or 17 or 18 or 19 or 20 |
| Outcome Measurement | 22 | exp treatment outcome/ |
|  | 23 | hospital management/ or hospital admission/ or hospital discharge/ or hospital readmission/ or hospital utilization/ or hospitalization/ |
|  | 24 | patient compliance/ or medication compliance/ |
|  | 25 | exp "quality of life"/ |
|  | 26 | exp mortality/ |
|  | 27 | daily life activity/ |
|  | 28 | ((clinical or treatment or patient? or surrogate) adj3 (outcome* or endpoint*)).ti,ab. |
|  | 29 | ((treatment* or drug* or medication* or clinical) adj2 (adherence or nonadherence or compliance or noncompliance or effectiveness or efficacy or failure? or response* or surveillance)).ti,ab. |
|  | 30 | ((adverse or safety or unintended or unintentional) adj2 (interaction? or response? or effect? or event? or reaction? or outcome?)).ti,ab. |
|  | 31 | (emergency adj2 (visit* or stay)).ti,ab. |
|  | 32 | (patient experience? or patient impact? or "length of stay" or "length of hospital stay" or complication? or health resource utili#ation or healthcare utili#?ation or hospitali#ation? or admission or readmission? or re-admission? or ED visit? or ER visit? or recovery or "quality of life" or QoL or "activities of daily living" or ADL or health status or disease progression or disease control or function* or survival or mental health outcome? or relapse* or symptomatic response).ti,ab. |
|  | 33 | 22 or 23 or 24 or 25 or 26 or 27 or 28 or 29 or 30 or 31 or 32 |
|  | 34 | 15 and 21 and 33 |
| Study Type | 35 | (comment or editorial or letter).pt. |
|  | 36 | 34 not 35 |
| Other | 37 | 36 not (exp animals/ NOT humans/) |
|  | 38 | limit 37 to (english language and yr="2000 -Current") |

PsycINFO Search

| **Concept** | **Layer** | **Search Terms** |
| --- | --- | --- |
| Digital Health Technology | 1 | digital technology/ or artificial intelligence/ or computer applications/ or electronic communication/ or mobile technology/ or navigation technology/ or wireless technologies/ |
|  | 2 | wearable devices/ |
|  | 3 | smartphone/ |
|  | 4 | (digital adj2 (tool? or device?)).ti,ab. |
|  | 5 | (wearable* adj2 (device? or sensor? or motion*)).ti,ab. |
|  | 6 | (wearable* adj2 (technolog* or monitor* or track*)).ti,ab. |
|  | 7 | (pedometer? or accelerometer?).ti,ab. |
|  | 8 | (mobile adj2 (technolog* or device?)).ti,ab. |
|  | 19 | (inertial adj2 (sensor? or device?)).ti,ab. |
|  | 10 | (ingestible adj2 (device? or technolog*)).ti,ab. |
|  | 11 | (activity adj2 (track* or monitor*)).ti,ab. |
|  | 12 | (smartphone? or (smart adj2 (phone? or device?))).ti,ab. |
|  | 13 | (Fitbit? or Apple watch* or Smartwatch* or ResearchKit? or HealthKit? or Healthpatch* or Garmen? or patient-facing technolog* or GPS system? or Global Positioning System? or mHealth technolog* or Textile piezoresistive sensor? or smartphone? or biochip? or digital implant? or implant technolog* or portable software or portable electronic app* or fitness track* or embedded sensor? or sensor technolog* or remote technolog* or wireless sensor? or motion track*).ti,ab. |
|  | 14 | 1 or 2 or 3 or 4 or 5 or 6 or 7 or 8 or 9 or 10 or 11 or 12 or 13 |
| Digital Health Data | 15 | (patient adj (monitor* or track* or generated*)).ti,ab. |
|  | 16 | (real world adj2 (evidence or data)).ti,ab. |
|  | 17 | (outcome? adj2 (measure* or predict*)).ti,ab. |
|  | 18 | (digital biomarker? or digital phenotyp* or ecological momentary assessment?).ti,ab. |
|  | 19 | 15 or 16 or 17 or 18 |
| Outcome Measurement | 20 | exp treatment outcomes/ |
|  | 21 | "treatment process and outcome measures"/ or patient reported outcome measures/ |
|  | 22 | health outcomes/ |
|  | 23 | "Recovery of Function"/ |
|  | 24 | patient admission/ or patient discharge/ or patient readmission/ |
|  | 25 | patient compliance/ or medication adherence/ |
|  | 26 | exp "quality of life"/ |
|  | 27 | "activities of daily living"/ |
|  | 28 | ((clinical or treatment or patient? or surrogate) adj3 (outcome* or endpoint*)).ti,ab. |
|  | 29 | ((treatment* or drug* or medication* or clinical) adj2 (adherence or nonadherence or compliance or noncompliance or effectiveness or efficacy or failure? or response* or surveillance)).ti,ab. |
|  | 30 | ((adverse or safety or unintended or unintentional) adj2 (interaction? or response? or effect? or event? or reaction? or outcome?)).ti,ab. |
|  | 31 | (emergency adj2 (visit* or stay)).ti,ab. |
|  | 32 | (patient experience? or patient impact? or "length of stay" or "length of hospital stay" or complication? or health resource utili#ation or healthcare utili#?ation or hospitali#ation? or admission or readmission? or re-admission? or ED visit? or ER visit? or recovery or "quality of life" or QoL or "activities of daily living" or ADL or health status or disease progression or disease control or function* or survival or mental health outcome? or relapse* or symptomatic response).ti,ab. |
|  | 33 | 20 or 21 or 22 or 23 or 24 or 25 or 26 or 27 or 28 or 29 or 30 or 31 or 32 |
|  | 34 | 14 and 19 and 33 |
| Study Type | 35 | (comment or editorial or letter).pt. |
|  | 36 | 34 not 35 |
| Other | 37 | 36 not (exp animals/ NOT humans/) |
|  | 38 | limit 37 to (english language and yr="2000 -Current") |

ClinicalTrials.gov

**Study Type:** All Studies

**Study Results:** Studies with Results

**Status: Recruitment:** Completed

**Results First Posted: From** 01/01/2000 **To** 02/14/2021

**Search (repeated in ‘Other terms’, and Targeted Search fields (‘Intervention/treatment’; ‘Title / Acronym’’ ‘Outcome Measure’):**

(wireless technology OR mobile app OR wearable OR fitness tracker OR smartphone OR digital technology OR sensor OR Fitbit OR Apple watch) AND (patient monitoring OR patient tracking OR real world data OR real world evidence OR digital biomarker)

EU Clinical Trials Register

**Trial Status**: Completed

**Date Range**: 2000-01-01 to 2021-02-14

**Results Status**: Trials with results

**Search 1**: "wireless technology" OR “mobile app” OR wearable OR "fitness tracker" OR smartphone OR "digital technology" OR sensor OR Fitbit OR "Apple watch"

**Search 2**: “patient monitoring” OR “patient tracking” OR “real world data” OR “real world evidence” OR “digital biomarker”
