# Supplementary material for: Digital Health Technology for Real-World Clinical Outcome Measurement Using Patient-Generated Data: Systematic Scoping Review
Source: J Med Internet Res. 2023 Oct 11;25:e46992. doi: 10.2196/46992 (PMC10600647; doi:10.2196/46992)
Supplement: Multimedia Appendix 4 [file jmir_v25i1e46992_app4.docx]

# Multimedia Appendix 4

## Geography


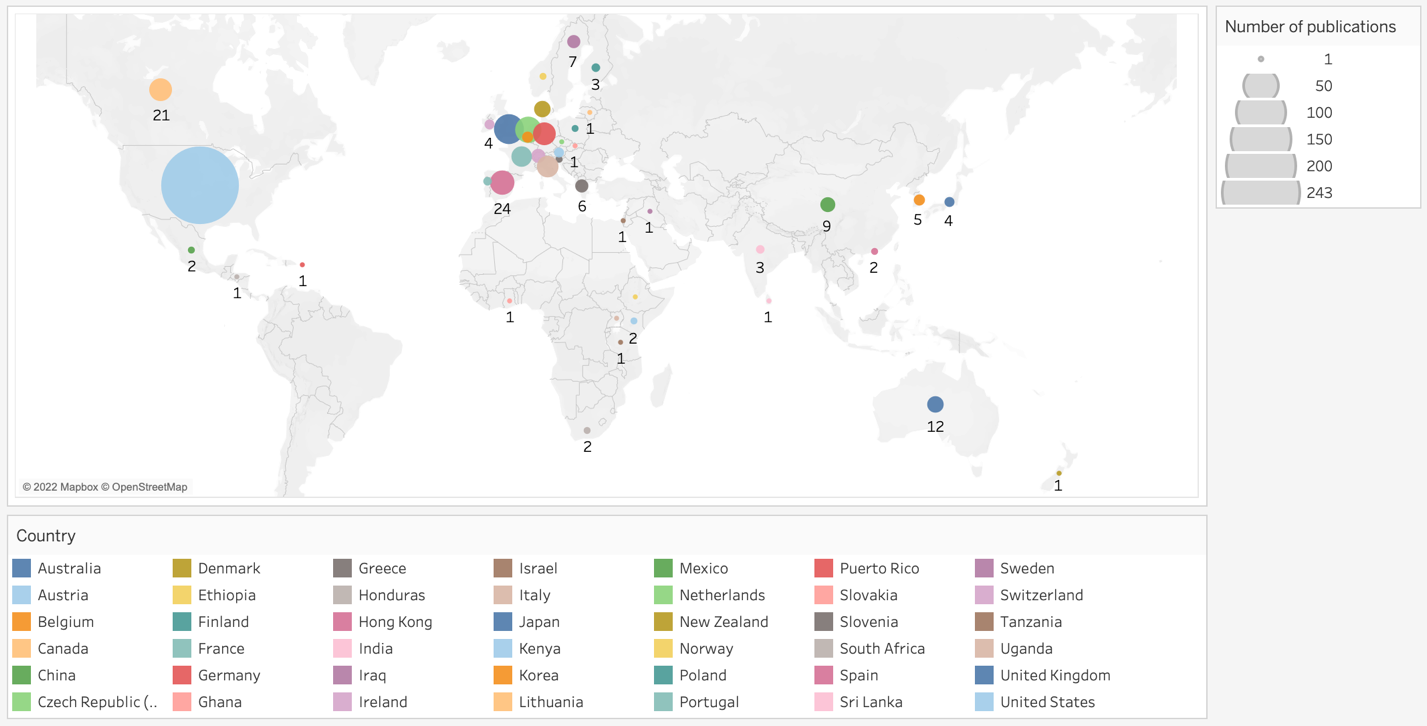


Figure S1. Geographical distribution of included studies by country of origin

## Disease Areas

Table S1. Summary of studies by disease and therapeutic area

| Therapeutic Area | Disease / Condition | Count | Proportion of all studies (%) |
| --- | --- | --- | --- |
| Audiology (n = 3) | | | |
|  | Acute coronary syndrome | 1 | 0.20% |
|  | Atrial fibrillation | 15 | 2.94% |
|  | Cardiac arrhythmias | 3 | 0.59% |
|  | Cardiac monitoring implant | 4 | 0.78% |
|  | Cardiac surgery | 2 | 0.39% |
|  | Catheter ablation for atrial fibrillation or bariatric surgery | 1 | 0.20% |
|  | Complete heart block | 1 | 0.20% |
|  | Coronary artery disease | 3 | 0.59% |
|  | Cardiovascular disease (general) | 4 | 0.78% |
|  | Fidelis lead fractures | 1 | 0.20% |
|  | Hearing loss | 1 | 0.20% |
|  | Heart failure | 13 | 2.55% |
|  | Hypertension | 16 | 3.14% |
|  | Ischemic heart disease | 2 | 0.39% |
|  | Myocardial infarction | 1 | 0.20% |
|  | Peripheral artery disease | 1 | 0.20% |
|  | Single ventricle | 1 | 0.20% |
|  | Syncope | 2 | 0.39% |
|  | Tinnitus | 2 | 0.39% |
| Dermatology (n = 2) | | | |
|  | Clinically atypical nevi | 1 | 0.20% |
|  | Melanoma | 1 | 0.20% |
| Eye Health (n=5) | | | |
|  | Maculopathy | 3 | 0.59% |
|  | Visual impairment | 2 | 0.39% |
| Gastroenterology (n = 7) | | | |
|  | Cirrhosis | 1 | 0.20% |
|  | Dyspepsia | 1 | 0.20% |
|  | Fecal incontinence | 1 | 0.20% |
|  | Gastroesophageal reflux disease | 2 | 0.39% |
|  | Irritable bowel syndrome | 2 | 0.39% |
| Hematology (n = 4) | | | |
|  | Allogeneic hematopoietic cell transplant | 1 | 0.20% |
|  | Pediatric blood and marrow transplant | 1 | 0.20% |
|  | Pediatric sickle cell disease | 1 | 0.20% |
|  | Sickle cell disease | 1 | 0.20% |
| Immunology (n = 6) | | | |
|  | Allergic rhinitis | 2 | 0.39% |
|  | Inflammatory bowel disease (IBD) | 4 | 0.78% |
| Infectious Diseases and Vaccines (n = 24) | | | |
|  | COVID-19 | 11 | 2.16% |
|  | Hepatitis C | 2 | 0.39% |
|  | Human immunodeficiency virus (HIV) | 7 | 1.37% |
|  | Malaria | 1 | 0.20% |
|  | Tuberculosis | 3 | 0.59% |
| Mental Health and Addictions (n = 111) | | | |
|  | Attention-deficit/hyperactivity disorder (ADHD) | 1 | 0.20% |
|  | Adverse posttraumatic neuropsychiatric sequelae (APNS) | 3 | 0.59% |
|  | Alcohol use disorder | 8 | 1.57% |
|  | Autism spectrum disorder | 2 | 0.39% |
|  | Bipolar disorder | 9 | 1.76% |
|  | Depression / major depressive disorder (MDD) | 16 | 3.14% |
|  | Eating disorders and disordered behaviour | 4 | 0.78% |
|  | Impulsive behaviour | 1 | 0.20% |
|  | Insomnia | 2 | 0.39% |
|  | Major depression disorder (MDD), bipolar I disorder, OR schizophrenia | 1 | 0.20% |
|  | Mental health diagnosis (MDD, PTSD, bipolar disorder, OR alcohol use disorder) | 1 | 0.20% |
|  | Mood disorders (general) | 1 | 0.20% |
|  | Obsessive-compulsive disorder (OCD) | 2 | 0.39% |
|  | Opioid/substance use disorder | 13 | 2.55% |
|  | Pediatric psychiatric diagnosis | 1 | 0.20% |
|  | Personality disorders | 2 | 0.39% |
|  | Postpartum depression | 1 | 0.20% |
|  | Psychosis and psychotic symptoms | 3 | 0.59% |
|  | Psychotic disorders (schizophrenia, schizoaffective disorder, bipolar disorder) | 7 | 1.37% |
|  | Schizophrenia | 25 | 4.90% |
|  | Self-injury | 1 | 0.20% |
|  | Serious mental illness | 3 | 0.59% |
|  | Suicidal attempt or ideation | 4 | 0.78% |
| Metabolism and Endocrinology (n = 45) | | | |
|  | Achilles tendon rupture | 1 | 0.20% |
|  | Chronic musculoskeletal pain | 1 | 0.20% |
|  | Diabetes (non-specific) | 8 | 1.57% |
|  | Diabetic polyneuropathy | 1 | 0.20% |
|  | Gaucher disease | 1 | 0.20% |
|  | Hypertension; Diabetes | 1 | 0.20% |
|  | Hyperthyroidism | 1 | 0.20% |
|  | Locomotive syndrome | 1 | 0.20% |
|  | Lower extremity impairments | 1 | 0.20% |
|  | Metatarsalgia | 1 | 0.20% |
|  | Overweight/obesity | 2 | 0.39% |
|  | Pectus carinatum | 1 | 0.20% |
|  | Spine disease | 1 | 0.20% |
|  | Thyrotoxicosis | 1 | 0.20% |
|  | Type 1 diabetes | 17 | 3.33% |
|  | Type 2 diabetes | 13 | 2.55% |
|  | Upper extremity illness | 1 | 0.20% |
| Neurology / CNS (n = 78) | | | |
|  | Amyotrophic lateral sclerosis (ALS) | 6 | 1.18% |
|  | Alzheimer's disease | 3 | 0.59% |
|  | Amnestic mild cognitive impairment | 2 | 0.39% |
|  | Ataxia-telangiectasia | 1 | 0.20% |
|  | Cerebral palsy | 5 | 0.98% |
|  | Concussion | 2 | 0.39% |
|  | Dementia and cognitive impairment | 4 | 0.78% |
|  | Duchenne muscular dystrophy | 2 | 0.39% |
|  | Epilepsy | 2 | 0.39% |
|  | Functional tremor | 1 | 0.20% |
|  | General neurocognition | 1 | 0.20% |
|  | Head trauma | 1 | 0.20% |
|  | Huntington's disease | 2 | 0.39% |
|  | Lewy body dementia | 1 | 0.20% |
|  | Multiple sclerosis | 9 | 1.76% |
|  | Neurodevelopmental disorders | 1 | 0.20% |
|  | Parkinson's disease | 16 | 3.14% |
|  | Spinal muscular atrophy | 1 | 0.20% |
|  | Stroke | 16 | 3.14% |
|  | Traumatic brain injury | 2 | 0.39% |
| Oncology (n = 36) | | | |
|  | Abdominal cancer | 1 | 0.20% |
|  | Brain tumor | 1 | 0.20% |
|  | Breast cancer | 6 | 1.18% |
|  | Cancer (multiple) | 2 | 0.39% |
|  | Cancer chemotherapy (multiple) | 4 | 0.78% |
|  | Cancer immunotherapy | 1 | 0.20% |
|  | Chemotherapy-induced peripheral neuropathy | 1 | 0.20% |
|  | Colorectal cancer | 1 | 0.20% |
|  | End-stage/palliative cancer | 3 | 0.59% |
|  | Gastrointestinal (GI) and lung cancer surgery | 1 | 0.20% |
|  | Genitourinary cancers | 1 | 0.20% |
|  | Gynecologic cancer surgery | 1 | 0.20% |
|  | Head and neck cancer | 5 | 0.98% |
|  | Hematologic malignancy | 2 | 0.39% |
|  | Malignant pleural mesothelioma | 1 | 0.20% |
|  | Metastatic solid tumors | 1 | 0.20% |
|  | Neuroendocrine tumors | 1 | 0.20% |
|  | Pancreatic surgery (pancreatic cancer or cysts) | 1 | 0.20% |
|  | Pediatric cancer | 1 | 0.20% |
|  | Radiation oncology | 1 | 0.20% |
| Other (n = 5) | | | |
|  | Globus sensation | 1 | 0.20% |
|  | Hypertension, heart failure, diabetes mellitus, tuberculosis, bipolar affective disorder, OR schizophrenia | 1 | 0.20% |
|  | Sleep apnea | 2 | 0.39% |
|  | Sleep disturbance | 1 | 0.20% |
| Pain (n = 14) | | | |
|  | Chronic low back pain | 2 | 0.39% |
|  | Chronic pain | 3 | 0.59% |
|  | Fibromyalgia | 3 | 0.59% |
|  | Migraine | 3 | 0.59% |
|  | Pain flares | 1 | 0.20% |
|  | Pain-related sleep disorders | 1 | 0.20% |
|  | Plantar fasciitis | 1 | 0.20% |
| Rehabilitation / Physical Therapy (n = 8) | | | |
|  | Anxiety and/or depression following traumatic brain injury | 1 | 0.20% |
|  | Concussion | 1 | 0.20% |
|  | Femoroacetabular impingement syndrome | 1 | 0.20% |
|  | Neuropathic diabetes | 1 | 0.20% |
|  | Posttraumatic headaches | 1 | 0.20% |
|  | Spinal cord injury | 1 | 0.20% |
|  | Use of knee-ankle-foot orthoses | 1 | 0.20% |
|  | Vestibular deficits | 1 | 0.20% |
| Respiratory / Pulmonary (n = 26) | | | |
|  | Asthma | 10 | 1.96% |
|  | Chronic obstructive pulmonary disease (COPD) | 5 | 0.98% |
|  | Chronic respiratory failure | 1 | 0.20% |
|  | Cystic fibrosis | 3 | 0.59% |
|  | Pediatric respiratory diseases | 5 | 0.98% |
|  | Pulmonary disease | 1 | 0.20% |
|  | Severe eosinophilic asthma | 1 | 0.20% |
| Rheumatology (n = 22) | | | |
|  | Idiopathic inflammatory myopathy | 1 | 0.20% |
|  | Juvenile arthritis | 3 | 0.59% |
|  | Lupus | 1 | 0.20% |
|  | Osteoarthritis | 3 | 0.59% |
|  | Rheumatic disease (general) | 2 | 0.39% |
|  | Rheumatoid arthritis | 12 | 2.35% |
| Surgery (n = 22) | | | |
|  | Colorectal surgery | 2 | 0.39% |
|  | Esophagectomy | 1 | 0.20% |
|  | Inguinal hernia | 1 | 0.20% |
|  | Joint surgery | 8 | 1.57% |
|  | Median Sternotomy | 1 | 0.20% |
|  | Nasal surgery | 1 | 0.20% |
|  | Pancreatectomy | 1 | 0.20% |
|  | Pediatric transplantation | 1 | 0.20% |
|  | Postoperative complications | 2 | 0.39% |
|  | Spinal surgery | 3 | 0.59% |
|  | Thoracoscopic surgery | 1 | 0.20% |
| Urology and Nephrology (n = 7) | | | |
|  | Incontinence | 3 | 0.59% |
|  | Kidney disease | 3 | 0.59% |
|  | Kidney transplant | 1 | 0.20% |
| Women's Health / Obstetrics and Gynecology (n = 6) | | | |
|  | Endometriosis | 1 | 0.20% |
|  | Heavy menstrual bleeding | 1 | 0.20% |
|  | Pregnancy | 2 | 0.39% |
|  | Pregnancy complication | 2 | 0.39% |
| **Total (n = 510)** | | | |
